# Genetic Variants in SNCA and the Risk of Sporadic Parkinson's Disease and Clinical Outcomes: A Review

**DOI:** 10.1155/2017/4318416

**Published:** 2017-07-11

**Authors:** Clarissa Loureiro das Chagas Campêlo, Regina Helena Silva

**Affiliations:** ^1^Memory Studies Laboratory, Department of Physiology, Universidade Federal do Rio Grande do Norte, Natal, RN, Brazil; ^2^Behavioral Neuroscience Laboratory, Department of Pharmacology, Universidade Federal de São Paulo, São Paulo, SP, Brazil

## Abstract

There is increasing evidence of the contribution of genetic susceptibility to the etiology of Parkinson's disease (PD). Genetic variations in the SNCA gene are well established by linkage and genome-wide association studies. Positive associations of single nucleotide polymorphisms (SNPs) in SNCA and increased risk for PD were found. However, the role of SNCA variants in individual traits or phenotypes of PD is unknown. Here, we reviewed the current literature and identified 57 studies, performed in fourteen different countries, that investigated SNCA variants and susceptibility to PD. We discussed the findings based on environmental factors, history of PD, clinical outcomes, and ethnicity. In conclusion, SNPs within the SNCA gene can modify the susceptibility to PD, leading to increased or decreased risk. The risk associations of some SNPs varied among samples. Of notice, no studies in South American or African populations were found. There is little information about the effects of these variants on particular clinical aspects of PD, such as motor and nonmotor symptoms. Similarly, evidence of possible interactions between SNCA SNPs and environmental factors or disease progression is scarce. There is a need to expand the clinical applicability of these data as well as to investigate the role of SNCA SNPs in populations with different ethnic backgrounds.

## 1. Introduction

Parkinson's disease (PD) is a neurodegenerative disorder that is characterized by motor dysfunction but also causes nonmotor deficits [1]. Although the etiology of PD remains unclear, the interaction between genetic and environmental factors has been implicated in the emergence of the disease [2, 3]. Genome-wide association studies (GWAS) have identified variants of many candidate genes that contribute to PD susceptibility, such as variations of the SNCA gene [4–6]. Moreover, certain polymorphisms of SNCA are among the major risk factors for sporadic PD [5].

The SNCA gene is located on human chromosome 4 and encodes the protein alpha-synuclein. The physiological function of alpha-synuclein is not completely understood. Studies have shown a key role for alpha-synuclein in the regulation of neurotransmitter release, synaptic function, and plasticity of dopaminergic neurons [7–9]. The involvement in dopaminergic transmission and the predominant presence of alpha-synuclein in Lewy bodies [10–12] denote the relationship of this protein with the etiology of PD. In addition, genetic data support the role of alpha-synuclein in the pathogenic process of the disease. For example, missense mutations in SNCA locus were identified in familial forms of PD (A53T, A30P, E46K, and H50Q) [13–17], as well as in sporadic PD patients (A18T and A29S) [18]. Further, duplications and triplications of the SNCA locus cause familial parkinsonism and correlate with disease severity [19, 20].

Single nucleotide polymorphism (SNP) analyses in case-control studies performed in different populations have shown an association between several SNCA polymorphisms and the risk of PD. For example, the dinucleotide repeat REP1 located in the SNCA promoter (SNCA-REP1) and the 3′ untranslated region (UTR) variants are frequently investigated [21]. Variations in these regions may increase susceptibility to PD by interfering with transcription factor binding sites [22, 23] and creating or destroying microRNAs target sites, which in turn modifies gene expression [24–26].

In spite of the fact that this type of study is frequent, investigations of the association between SNPs and the risk of PD in different populations show conflicting results. Furthermore, the consequences of genetic variability on clinical phenotypes, as well the interaction between genetic and environmental substrates, are poorly elucidated. Ideally, data from SNP studies would improve knowledge of pathophysiological pathways and help to target the best therapeutic program. This review aims to identify and compare the main SNP association studies conducted in different populations. The role of SNCA polymorphisms as a risk factor for PD and their association with clinical outcomes are discussed.

## 2. Literature Search

We conducted a survey in the relevant databases PubMed/Medline and Scopus for studies up to July 2016 using combinations of the keywords “polymorphism”, “alpha synuclein”, “SNCA gene”, and “Parkinson's disease”. We selected articles that met the following inclusion criteria: (a) articles that are written in English; (b) articles that described investigations of SNPs in the SNCA gene; (c) articles that had available data of allele and genotype distributions; (d) studies that were conducted in humans; (e) studies including participants who were diagnosed with Parkinson's disease. 57 studies were selected to conduct the review. In addition, despite not necessarily meeting the selection criteria, other relevant publications were included throughout the article in order to foment discussion.

The following information was extracted from each study: authors, year of publication, country of the studied population, number of patients and control subjects, risk association with PD, and investigation of environmental factors and clinical outcomes. The association of SNCA polymorphisms and PD susceptibility was often assessed by comparing the frequency of risk allele and risk genotypes in patients and controls. Values of odds ratio (OR) and confidence interval (95% CI) indicated the characteristic and significance of the association (OR values higher than 1 indicated increased risk and values lower than 1 suggested reduced risk).

Table 1 summarizes the main data from the 57 selected studies. The majority of the studies were performed in Caucasian and Asian populations. The studies were carried out in fourteen different countries (Mexico, China, Russia, Germany, Taiwan, USA, Japan, Spain, Italy, Ireland, Netherlands, Norway, Australia, and Greece) assembling patients from twenty countries (Mexico, China, Russia, Germany, Taiwan, USA, Japan, Spain, Italy, Norway, Netherlands, Serbia, Ireland, Australia, France, Greece, Poland, Sweden, Iran, and Australia). Sample sizes varied from 91 to 5302 patients in PD groups. The mean age at onset of PD varied from 45.2 to 68.2 years old.

## 3. SNPs and the Risk for Parkinson's Disease

**Figure 1:**
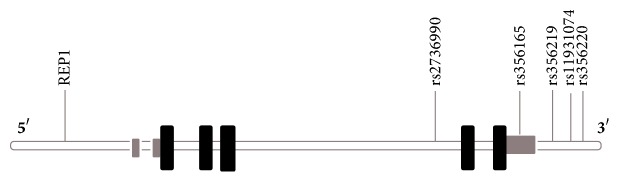
Diagram illustrating the locations of the main SNPs in human SNCA gene reviewed in the present study: SNCA-REP1 (promoter region), rs2736990 (intron 4), rs356165 (3′ UTR), and rs356219, rs356220, and rs11931074 (3′ end). Black boxes indicate translated exons, grey boxes indicate untranslated regions, and the white line indicates introns.

GWAS have identified 28 distinct loci that modify the individual risk to PD [85] and suggest that genetic factors contribute to at least one-fourth of the total variation in liability to PD [86]. The two most consistent genes for susceptibility to sporadic PD are the alpha-synuclein (SNCA) and microtubule-associated protein tau (MAPT) genes, which can exert independent or joint effects on the risk of PD [56, 59, 61, 62, 87]. In addition, variants in other genes previously linked with autosomal forms (LRRK2, PARK16–18, and GBA) have also shown association with PD risk [64, 88–90]. Based on odds ratio and confidence intervals values in the selected studies, thirty-nine different SNPs in the SNCA gene showed a statistically significant effect on PD susceptibility: nine variants in the 5′ end, nine variants near the 3′ end, and 25 intron variants (Table 2). The locations of the six more frequently investigated SNPs within the SNCA gene are illustrated in Figure 1.

Two major linkage disequilibrium blocks in the SNCA gene have been proposed: (1) a 5′ block that extends from the promoter-enhancer region to exon 4 and (2) a 3′ block that includes intron 4, 3′ UTR, and the 3′ end region of the gene [73, 80]. SNPs in the 3′ block presented a more expressive association with PD. The largest number of significant markers in the 3′ block across different populations suggests a major causal effect for variants located in the 3′ end compared with the 5′ end. The 3′ block contains elements with higher conservation across species, which emphasize its biological relevance [25].

The polymorphic microsatellite REP1 (D4S3481) in the promoter region of SNCA is one of the most frequently investigated polymorphisms and was pointed out as a risk factor in thirteen of the articles [47, 50, 60, 64, 65, 68, 71–73, 75, 77, 79, 81]. REP1 region plays a crucial role in the regulation of alpha-synuclein protein expression. Variants in REP1 are the only putative functional polymorphism identified within the SNCA locus. The REP1 SNP is a triallelic polymorphism with the longest (263 bp) and intermediate length (261 bp) alleles usually associated with an increased risk for PD, as seen in studies with North American [47, 50, 60, 64, 65, 68, 71, 72, 75, 81], German [73], Greek [79], and Dutch [77] samples. In 2006, a meta-analysis including over 5000 subjects provided strong evidence that the 263 bp allele was more frequent in cases and that the 259 bp allele was more frequent in controls (indicating decreased risk of PD) [91]. The other four SNPs in the 5′ region (rs2619362, rs2619363, rs2619364, and rs2583988) contributed to the increase in the risk of PD in North American [49, 65] and European [56, 61, 77] populations.

Twenty-five intronic variants were associated with PD susceptibility, although the precise function of those variants of human SNCA is still unknown. The SNP rs2736990 in intron 4 was the most frequent, with the exception of one study [40]; this SNP was a consistent risk factor for PD [27, 28, 41, 49, 52, 55]. Similarly, the SNPs rs2572324, rs7684318, and rs894278 also increased susceptibility to sporadic PD [49, 60, 64, 65]. In contrast, a significant reduction of PD risk was found for the SNP rs356186 in Italian [56], North American [60], and Irish [61] samples.

Recently, the SNPs located near the 3′ end have also been identified as risk factors for PD. Within the 3′ region, the majority of the studies focused on rs356219, rs11931074, and rs356165. Among them, the SNP rs356219 was the most investigated, and it stands out as a consistent risk factor for PD in twelve of the studies. Variants in the 3′ region possibly increase SNCA expression because of the misregulation of the posttranscriptional control. In the 3′ untranslated region (UTR), there are target binding sites for two microRNAs (mir-7 and mir-153), and alterations in these regions might affect mRNA stability and translation [92]. Further, mir-7 and mir-153 were associated with SNCA mRNA expression in human studies [23, 24]. The SNP rs356219 showed a robust association as a common susceptibility marker in twelve studies with patients from Russia [44], Germany [46, 80], Spain [58, 59], Japan [52], China [53], USA [28, 65], United Kingdom [78], and Netherlands [73]. Similarly, the SNP rs11931074 showed a significant association with increased PD risk in seven studies, four of which were performed in Chinese samples [32, 34, 39, 40].

The variants rs356221, rs356165, and rs356182 in 3′ UTR also contribute significantly to the increase in the PD risk in Germany [51, 77, 80], USA [28, 50], China [34], Taiwan [45], and Netherlands [73]. These findings reinforce the role of a posttranscriptional mechanism in PD etiology. The variant rs356165 was the most expressive [28, 50, 51, 54, 61, 73, 77, 80].

## 4. SNCA SNPs and Environmental Factors Interactions

Despite the relevance of environmental data to the understanding of the etiology of PD, the majority of studies contain little or no information on possible associations between SNCA SNPs and environmental factors (Table 3).

Sporadic PD is considered a result of complex interactions between genetic and environmental risk factors. Professional pesticide exposure, rural living, and well-water drinking were reported to increase the risk of PD [93–95]. In contrast, cigarette smoking [96, 97] and caffeine intake [98] have been pointed out as protective factors. Furthermore, though less consistently, reduced PD risk was associated with alcohol drinking [99]. Potential interactions between SNCA SNPs and pesticide exposure, smoking habits, head injury, or coffee and alcohol drinking were investigated in a few of the studies [47, 50, 52, 55, 56]. Overall, data demonstrated some pairwise interactions, but without reaching significant levels after Bonferroni correction. For example, although Miyake et al. [52], Chung et al. [47], and Gao et al. [55] found a significant protective effect of smoking habits against PD, corroborating epidemiological data, only the first study found significant interactions between SNPs rs356219 and rs356220 and smoking.

Meta-analyses [96, 97] have reinforced the inverse association between cigarette smoking and PD risk more consistently than other environmental factors (rural living, well-water consumption, farming, and the use of pesticides). One of the mechanisms suggested to underlie this neuroprotection is the reduction of brain levels of the enzyme monoamine oxidase B (MAO B), an isoform that selectively metabolizes dopamine. This reduction would enhance the levels of dopamine and decrease the production of hydrogen peroxide and oxidative stress rates [100]. An alternative explanation is that smoke induces cytochrome P-450 enzyme activity. This enzyme is responsible for the metabolism of antipsychotic drugs and the detoxification of certain environmental toxins such as MPTP [101]. In this respect, a case-control study carried out in European countries investigated polymorphisms with relevance to brain expression and metabolism of substances contained in tobacco smoke and confirmed significant interactions of SNPs in cytochrome P-450 enzyme family genes (GSTM1, GSTP1, and NAT2). However, there is no information about the mechanisms that explain the biological interaction between SNCA genotypes and cigarette smoking.

## 5. SNCA SNPs and PD Clinical Outcomes Interactions

The influence of polymorphisms on PD phenotypic variability remains unclear. Indeed, few studies specifically investigated their associations with particular aspects of the disease, such as disease history and clinical outcomes (Table 4).

The age at onset is the most frequently investigated and well established aspect of disease history. Polymorphisms located in the promoter (REP1), introns (rs2736990, rs894278), and 3′ region (rs356219 and rs356165) are suggested to be predictors of earlier PD onset in Australian and Chinese [35], Spanish [54], German [46], and UK [61] samples. The identification of genetic features associated with the onset of PD has a relevant potential for therapeutic targeting. In view of the fact that neurodegeneration precedes the appearance of motor symptoms [102], it is critical to predict the risk of developing PD prior to clinical manifestations.

Even though it is expected that SNCA variants would influence individual traits or phenotypes of sporadic PD, this association was poorly explored in the studies. In a longitudinal study with a North American sample, Ritz et al. [50] showed that the REP1-263 bp promoter variant and the G-rs356165 allele are risk factors to faster motor progression in Caucasian and non-Caucasian PD patients (OR 1.66; 95% CI: 0.96–2.88). Wang et al. [31] found a protective association between T-rs11931074 allele and motor severity. However, in a larger sample of Caucasian patients, Markopoulou et al. [43] showed that REP1-263 pb allele reduced the risk of developing motor impairments. This divergence can be a result of differences in the methods of motor evaluation. According to Markopoulou et al. [43], the discrepancies may also suggest a dual and time-dependent role of SNCA.

Impairments in the olfactory function are a common early-stage nonmotor feature of PD, and the TT-rs11931074 genotype may increase the risk of hyposmia in PD cases [34].

Few other studies pointed out weak or absent associations with clinical outcomes such as anxiety or depression [32, 34, 37, 40, 74], sleep and autonomic disorders [34, 74], and cognitive impairments [31, 32, 34, 37, 40, 74]. Regarding cognitive features, Markopoulou et al. [43] demonstrated that the REP1-259 pb allele increased the risk of cognitive outcomes. Trotta et al. [56] found significant associations of C-rs10018362, T-rs7689942, and G-rs1348224 alleles with PD with dementia and also identified a specific haplotype in intron 4 of SNCA (C-rs62306323 and T-rs7689942) associated with increased risk of PD with dementia. It is important to mention that limited data of SNP associations with PD phenotype in cross-sectional studies might be related to a single event of clinical assessment, which could mask possible effects. Studies of such associations could provide important clinical applicability of the significant findings of genotyping studies. More studies evaluating specific clinical aspects, especially with longitudinal designs, are necessary to enhance the understanding of how genetic factors contribute to PD.

## 6. Biological Effects of SNCA SNPs

There is increasing evidence supporting the biological effects of genetic variants in SNCA, possibly by modifying alpha-synuclein expression. Among the studies listed in Table 5, the associations of SNCA-REP1 and 3′ variants (rs356219 and 11931074) and peripheral alpha-synuclein levels were investigated in Chinese [57] and North American [65] populations. Mata et al. [65] found an association of CC-356219 genotype with increased plasma levels of alpha-synuclein. A variation in the REP1 region might affect transcriptional activity by increasing the expression of alpha-synuclein and consequent protein accumulation [22, 23]. SNCA duplication and triplication in familial PD have been linked to increased mRNA expression levels [103] and to disease severity [19, 20].

In postmortem brain tissue studies, the 3′ region SNPs rs356219 [84], rs356165 [24], and rs11931074 [39] have been associated with increased gene expression. For rs356219, the heterozygote CT genotype correlated with higher levels of SNCA-mRNA in the substantia nigra of PD patients. However, the TT protective genotype was accompanied by higher expression in the cerebellum, a structure that is more preserved in the course of PD [84]. Similarly, G-rs356219 allele carriers presented higher levels of the SNCA112-mRNA isoform in the frontal cortex [24]. Further, PD patients presented higher levels of the SNCA-112 and SNCA-98 transcripts in the cerebellum and occipital cortex when compared to controls [39].

Linnertz et al. [104] investigated the effects of SNCA SNPs in 5′ and 3′ regions on SNCA expression in postmortem brains from neurologically normal subjects. For REP1, the 256 bp/256 bp genotype correlated with lower SNCA-mRNA levels, corroborating the hypotheses that decreased SNCA levels protect against the disease. Unexpectedly, the protective genotypes AA-rs356219 and AA-rs365165 in the 3′ region correlated with higher levels of SNCA-mRNA in the temporal cortex and substantia nigra, which highlights an expanded regulatory effect of this region on total SNCA-mRNA levels.

Regarding in vivo studies, patients with SNP rs2583988 genotypes did not present alterations in alpha-synuclein levels in peripheral blood mononuclear cells, while the protein levels were reduced in the absence of the REP1 risk allele [84]. For SNPs in the 3′ region, while carriers of T-rs11931074 allele presented reduced levels of protein in serum [57], a higher level of alpha-synuclein in plasma was observed in carriers of the C-rs356219 allele [65].

## 7. Genetical Background, Ethnicity, and the Effect of SNCA SNPs

Methodological aspects such as variations in the sample size, control of population stratification, and statistical analyses can explain the discrepancies in the effects of SNPs among the different studies. In addition, it is common to exclude the influence of confounder variables as sex, age, and ethnic background, which can modify the results substantially.

The majority of the studies were carried out in countries of Europe, North America, and Asia, that is, populations with mainly Caucasian and Asian genetic backgrounds. Despite the fact that ethnic differences can preclude the generalization of the results in genetic studies, many of the SNPs showed similar effects in groups with different genetic backgrounds. For example, the SNP rs356219 remained an important risk factor for PD in studies performed within North American [65], Spanish [63], Russian [44], and Chinese [41] populations. Nevertheless, given the significance of results and perspectives of clinical advancement, it seems necessary to extend these investigations to other continents, in order to confirm whether these genetic effects are consistent across different populations and to verify the implications of between-population heterogeneity.

Finally, apart from variants in the SNCA gene, it is necessary to consider the role of interactions between multiple genetic variants in disease risk and clinical profile. For example, investigations of phenotypic diversity in PD have identified an association between SNCA and MAPT. This association contributed to increased cognitive and motor severity in a Chinese sample [31] and influenced the development of cognitive impairment and dementia in a British sample [78]. In addition, polymorphisms in GAB [28] and LRRK2 [58] have been associated with earlier age at the onset in a European-American population and in a Spanish sample, respectively. Also, in North Americans, APOE variants predicted lower cognitive performance in PD patients [42].

## 8. Conclusions

This review collected the contributions of polymorphisms in the SNCA gene to PD susceptibility and clinical phenotypes. In most of the studies, the influence of polymorphisms in multiple regions of SNCA gene was pointed out, such as the promoter region (REP1-SNCA), 3′ end (e.g., rs11931074 and rs356219), 3′ untranslated regions (e.g., rs356165), and introns (e.g., rs7684318, rs894278, and rs276990). In addition, we highlight that it is necessary to expand the clinical applicability of these data, as well as investigate the role of SNCA variations in populations with different ethnic backgrounds.

## Figures and Tables

**Table 1:** Characterization of the reviewed studies.

Studies	Study ID	Country of participants	Sample size		Onset age
			PD	Ctr	(mean ± SD)
Davila-Ortiz de Montellano et al., 2016	[27]	Mexico	171	171	—
Davis et al., 2016	[28]	USA	418	150	60.4 ± 11.1
García et al., 2016	[29]	Mexico	106	135	56.2 ± 14.4
Shahmohammadibeni et al., 2016	[30]	Iran	489	489	—
Wang et al., 2016	[31]	China	296	—	—
Cheng et al., 2016	[32]	China	1053	1152	52.0 ± 10.4
Guella et al., 2016	[33]	Multicentric^*∗*^	1492	971	60.3 ± 10.2
Chen et al., 2015	[34]	China	218	110	60.6 ± 07.4
Huang et al., 2015	[35]	China, Australia	402	—	—
Han et al., 2015	[36]	China	91	92	—
Chen et al., 2015	[37]	China	1276	846	56.3 ± 11.5
Gao et al., 2015	[38]	USA	507	1330	68.3 ± 5.8
Cardo et al., 2014	[39]	China	752	489	—
Guo et al., 2014	[40]	China	1011	721	56.6 ± 11.8
Pan et al., 2013	[41]	China	515	450	45.2 ± 04.6
Mata et al., 2014	[42]	USA	1191	—	59.46 ± 10.6
Markopoulou et al., 2014	[43]	USA	1098	—	62.2
Emelyanov et al., 2013	[44]	Russia	244	308	—
Wu-Chou et al., 2013	[45]	Taiwan	626	473	63.2 ± 07.8
Brockmann et al., 2013	[46]	Germany	1396	—	56.9 ± 01.9
Chung et al., 2013	[47]	USA	1098	1098	60.4
Pihlstrøm et al., 2013	[48]	Norway and Sweden	1380	1295	59.0
Heckman et al., 2012	[49]	USA	426	769	62.0 ± 12.0
Ritz et al., 2012	[50]	USA	232	—	—
Schmitt et al., 2012	[51]	Germany	980	1005	59.4 ± 12.2
Miyake et al., 2012	[52]	Japan	229	357	65.7 ± 08.8
Pan et al., 2012	[53]	China	403	315	57.8 ± 08.6
Cardo et al., 2012	[54]	Spain	727	480	—
Gao et al., 2012	[55]	USA	584	1571	68.2 ± 05.7
Trotta et al., 2012	[56]	Italy	904	891	56.1 ± 11.0
Hu et al., 2012	[57]	China	110	136	56.7 ± 10.8
Botta-Orfilla et al., 2012	[58]	Spain	84	—	—
Mata et al., 2011	[59]	Spain	1445	1161	60.0 ± 12.2
Chung et al., 2011	[60]	USA	1103	1103	62.2
Elbaz et al., 2011	[61]	Multicentric^*∗∗*^	5302	4161	—
Wider er al., 2011	[62]	USA, Ireland, Norway	1020	1095	58.0 ± 12.0
Botta-Orfilla et al., 2011	[63]	Spain	757	708	—
Biernacka et al., 2011	[64]	USA	1098	1098	62.2
Mata et al., 2010	[65]	USA	1956	2112	58.7 ± 11.9
Yu et al., 2010	[66]	China	332	300	54.3 ± 11.1
Hu et al., 2010	[67]	China	330	300	52.6 ± 11.8
Gatto et al., 2010	[68]	USA	333	336	—
Rajput et al., 2009	[69]	Canada	452	245	—
Sutherland et al., 2009	[70]	Australia	331	296	60.1 ± 10.6
Brighina et al., 2009	[71]	USA	893	893	62.1
Kay et al., 2008	[72]	USA	1802	2192	—
Myhre et al., 2008	[73]	Netherlands	236	236	—
Verbaan et al., 2008	[74]	Netherlands	295	150	—
Brighina et al., 2008	[75]	USA	833	833	61.9
Ross et al., 2007	[76]	Ireland	186	186	50.0 ± 11.0
Winkler et al., 2007	[77]	Germany, Serbia	397	270	—
Goris et al., 2007	[78]	UK	659	2176	63.0
Hadjigeorgiou et al., 2006	[79]	Greece	178	186	63.3 ± 9.6
Mueller et al., 2005	[80]	Germany	669	1002	55.4 ± 19.1
Mamah et al., 2005	[81]	USA	557	557	63.0
Spadafora et al., 2003	[82]	Italy	186	182	—
Izumi et al., 2001	[83]	Japan	200	250	61.0 ± 09.1

ID: identification; PD: Parkinson's disease group; Ctr: control group; SD: standard deviation. ^*∗*^The countries of origin of participants were New Zeeland, Canada, UK, and USA. ^*∗∗*^The countries of origin of participants were Australia, France, Germany, Greece, Ireland, Italy, Norway, Poland, Sweden, and USA.

**Table 2:** Single nucleotide polymorphisms (SNPs) in SNCA associated with Parkinson's disease.

Variant	Region	Alleles	Studies^*∗*^ with significant association with PD risk
REP1	Promoter	259 bp/261 bp/263 bp	[47, 50, 60, 64, 65, 68, 71–73, 75, 77, 79, 81] (↑) [43, 56, 76] (↓)
rs1372519	Promoter	A/G	[49] (↓)
rs2301134	Promoter	C/T	[45, 76] (↑)
rs2301135	Promoter	C/G	[45] (↓)
rs2619361	Promoter	A/C	[49] (↑)
rs2619362	5′ region	C/T	[49] (↑)
rs2619363	5′ region	G/T	[49, 77] (↑)
rs2619364	5′ region	A/G	[66, 77] (↑)
rs2583988	5′ region	C/T	[49, 56, 61, 77] (↑)
rs2119787	Intron	A/G	[65] (↓)
rs2197120	Intron	A/G	[56] (↓)
rs2572324	Intron	C/T	[49, 60, 64, 65] (↑)
rs2583959	Intron	C/G	[49, 64] (↑)
rs2736990	Intron	T/C	[27, 28, 41, 47, 49, 52, 55, 60] (↑) [40] (↓)
rs2737020	Intron	C/T	[76] (↓)
rs2737029	Intron	C/T	[56, 65, 80] (↑)
rs2737033	Intron	A/G	[46] (↑)
rs356164	Intron	C/G	[76] (↓)
rs356168	Intron	A/G	[64, 80] (↑)
rs356186	Intron	A/G	[56, 60, 76] (↓)
rs356203	Intron	A/G	[27, 80] (↑)
rs356204	Intron	A/G	[80] (↑)
rs3822086	Intron	C/T	[37] (↓)
rs3857057	Intron	A/G	[33] (↑)
rs3857059	Intron	A/G	[29, 80] (↑)
rs6848726	Intron	C/T	[80] (↓)
rs7684318	Intron	C/T	[27, 66] (↑)
rs7689942	Intron	C/T	[33] (↑)
rs894278	Intron	G/T	[35, 36] (↑)
rs1372520	Intron	C/T	[47, 49] (↑) [60] (↓)
rs10018362	Intron	C/T	[33] (↑)
rs2737012	Intron	C/T	[49] (↑)
rs3756063	Intron	C/G	[36] (↑)
rs3775423	Intron	C/T	[48, 60, 64] (↑)
rs356221	3′ region	A/T	[45, 48, 77] (↑)
rs356165	3′ region	A/G	[28, 50, 51, 54, 69, 73, 76, 77, 80] (↑)
rs356182	3′ region	A/G	[32] (↑)
rs356218	3′ region	A/G	[47, 49, 60] (↑)
rs356219	3′ region	A/G	[28, 42, 44, 46, 52, 53, 59, 61–63, 65, 73, 78, 80] (↑)
rs356220	3′ region	C/T	[27, 28, 30, 33, 52, 55, 80] (↑) [40] (↓)
rs356225	3′ region	C/T	[33] (↑)
rs181489	3′ region	C/T	[61, 76, 77] (↑)
rs11931074	3′ region	G/T	[30, 31, 34, 39, 55, 57, 61] (↑) [37, 45] (↓)

SNP: single nucleotide polymorphism. ^*∗*^See Table 1. Arrows indicate whether the SNP increased (↑) or reduced (↓) PD susceptibility in each study, based on values of odds ratio and confidence intervals.

**Table 3:** Association of environmental factors of PD with SNPs in SNCA.

Environmental factors	Studies^*∗*^	Results
Smoking	[34, 38, 47, 50, 52, 55, 56, 68, 70]	Individuals with GG-rs356219 and TT-rs356220 genotypes [52] and carriers of allele REP1-263 bp [47] who never had smoked had a significantly increased risk of PD.

Coffee intake	[38, 47, 55, 56]	No SNPs were significantly associated with this factor.

Pesticide exposure	[47, 68, 70, 71]	Chung et al. [47] found a suggestive association between C-rs3775423 and pesticide exposure.

Alcohol drinking	[47, 75]	Brighina et al. [75] found independent effects to alcohol consumption and REP1-SNCA. Alcohol use was associated with a decreased PD risk.

Head injury	[38]	Head injury increases PD risk, especially when it happens before the age of 30; no significant association with SNCA SNPs.

^*∗*^See Table 1.

**Table 4:** Association of clinical aspects of PD and SNPs within SNCA.

Variables	Studies^*∗*^	Results
Age at onset	[28, 32, 35, 37, 40, 41, 46, 53, 54, 58, 66, 69, 70, 72, 79]	SNPs were associated with earlier PD onset: REP1-263 bp [35, 69, 79]; C-rs2736990 [41]; G-rs894278 [35]; G-rs356219 [46, 53, 58]; G-rs356215 [54]

Motor outcomes^a^	[28, 31, 32, 34, 43, 50, 51, 74]	Wang et al. [31] found a protective association between T-rs11931074 allele carriers and motor severity. Ritz et al. [50] found that REP1-263 bp and G-rs356165 alleles increased the risk of faster decline of motor function, whereas Markopoulou et al. [43] demonstrated that REP1-263 bp allele reduced the risk of developing motor impairments

Cognition outcomes^b^	[31–34, 37, 40, 42, 43, 74, 78]	Markopoulou et al. [43] showed an increased risk of cognitive impairment in carriers of REP1-259 bp allele. Guella et al. [33] found a significant association of C-rs10018362, T-rs7689942, and G-rs1348224 alleles with PD with dementia

Anxiety and depression^c^	[32, 34, 37, 40, 74]	No SNPs were significantly associated with symptoms

Autonomic and sleep disorders^d^	[34, 74]	No SNPs were significantly associated with symptoms

Hyposmia	[34, 74]	In Chen et al.'s study [34], TT-rs11931074 genotype increased the risk of hyposmia in PD

^*∗*^See Table 1. ^a^Questionnaires used: Unified Parkinson Disease Rating Scale-III and Hoehn and Yahr. ^b^Questionnaires used: Mini-Mental State Examination, Frontal Assessment Battery, Montreal Cognitive Assessment, Scales for Outcomes in Parkinson's Disease Cognition, Hopkins Verbal Learning Test-Revised, Letter-Number Sequencing Test and Trail Making Test, Semantic and Phonemic Verbal Fluency Tests, and Benton Judgment of Line Orientation test. ^c^Questionnaires used: Hamilton Rating Scale for Depression and Anxiety and Beck Depression Inventory. ^d^Questionnaires used: REM Sleep Behavior Disorder Questionnaire, and Scales for Outcomes in Parkinson's Disease Autonomic, Nighttime Sleep, Daytime Sleepiness and Psychiatric Complications.

**Table 5:** Interactions between alpha-synuclein levels and SNCA SNPs.

Study	SNP	Risk allele	Results
Hu et al. [57]	rs11931074	T	The allele was associated with reduced levels of alpha-synuclein in serum
	REP1		Different alleles and genotypes did not influence levels of alpha-synuclein in serum

Mata et al. [65]	rs356219	C	CC genotype was correlated with increased levels of alpha-synuclein in plasma

Fuchs et al. [84]^a^	rs2583988	T	No correlation with alpha-synuclein mRNA or protein levels
	REP1	261 pb	256 pb/256 bp genotype was associated with lower alpha-synuclein levels assessed in blood samples; no effect in brain samples
	rs356219	C	CT genotype correlated with higher SNCA mRNA levels in the substantia nigra and TT genotype showed higher SNCA mRNA levels in the cerebellum; no effect in blood samples

McCarthy et al. [24]^a^	rs2736990	G	In the 3 SNPs, the GG genotype correlated with an increased expression ratio of SNCA112 mRNA in the frontal cortex
	rs356165	G	
	rs356219	G	

McCarthy et al. [24]^a^	rs3857059	G	No correlation between genotypes and the ratio expression levels of SNCA112 mRNA
	rs17016074	A	

Cardo et al. [39]^a^	rs356165 rs11931074	G T	No significant differences for the SNCA isoform levels between the different genotypes assessed in brain tissues

^a^Postmortem studies.
